# Scars of stroke care emerge as COVID-19 shifts to an endemic in many countries

**DOI:** 10.25122/jml-2022-1005

**Published:** 2022-05

**Authors:** Diana Alecsandra Grad, Razvan Mircea Chereches, Stefan Strilciuc, Dafin Muresanu

**Affiliations:** 1. RoNeuro Institute for Neurological Research and Diagnostic, Cluj-Napoca, Romania; 2. Department of Public Health, Babes-Bolyai University, Cluj-Napoca, Romania; 3. Department of Neurosciences, Iuliu Hatieganu University of Medicine and Pharmacy, Cluj-Napoca, Romania

COVID-19 has provoked a significant shift in health services delivery worldwide by limiting access to patients suffering acute episodes in need of immediate care [1, 2] and with chronic diseases [3], disrupting essential preventive efforts (*i.e*., immunization [4] and screening programs [5]), increasing burnout levels among health professionals (residents or senior staff) [6–8] and concentrating primary care providers in COVID-19-related surveillance and control efforts [9].

Stroke hospitalization rates (for ischemic [10] and hemorrhagic [11] stroke and transient ischemic attack (TIA) [2]) have been affected by the ongoing pandemic. Emergency assistance [12, 13] is a mandatory component of the stroke care protocol needed to increase survival rates. However, with multiple media reports [14, 15] pointing toward the growing cases of COVID-19 hospital outbreaks [16] (that affected hospitalized patients, hospitalization rates, and attending health professionals [17, 18]), fear of getting infected in the hospital was one of the causes of low admission for stroke rates. Other factors impairing hospital care and neurorehabilitation regimens were pre-and within-pandemic changes for shift patterns and the length of stay influenced by COVID-19 medical needs and the preventive measures [19].

The number of patients diagnosed and treated for stroke decreased in the first and second years of the pandemic. There were 25.3%, respectively 26.7% fewer thrombectomies and thrombolysis performed and 40% fewer stroke-related admissions in February 2020 compared to February 2019 in China [20]. In Spain, weekly stroke admissions reported for public hospitals from the North-West region reported lower statistically significant numbers (ranging from 14.5% to 6.5%). At the same time, in referral centers from other autonomous communities, results showed only slight changes (Cantabria and Navarra, of 0.5% and 2.5%) [10]. In Germany, declines in stroke cases have been more accentuated (17.4% for acute ischemic stroke and over 22% for TIA) [21]. In Qatar, the cases decreased by almost half (n=630) in the first three months of the pandemic [22]. A global cross-sectional analysis involving 70 countries and 403 countries analyzed stroke cases from November 2019 until June 2020 reported that the highest decrease in stroke hospitalizations was observed in Africa (30.2%), followed by North (18.8%) and South America (17.8%) [23]. In Europe, the number of stroke diagnoses dropped by 10.9%. As for intravenous thrombolysis, pandemic changes ranged from 24.2% (South America) and 9.9% (Asia).

**Figure 1:**
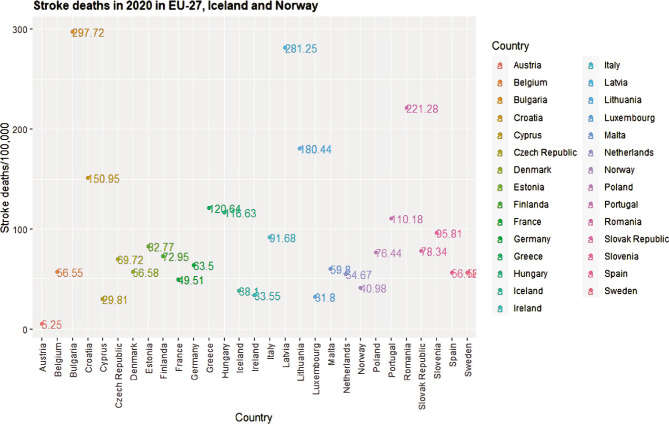
Number of deaths per 100,000 inhabitants computed using the crude numbers reported in individual Country Health Profiles for 2020 and country population retrieved from Worldometer [25].

**Figure 2:**
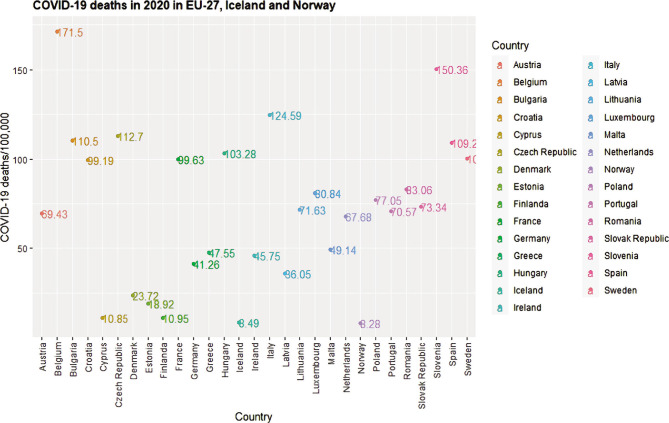
Number of deaths per 100,000 inhabitants computed using the crude numbers reported in individual Country Health Profiles for 2020 and country population retrieved from Worldometer [25].

In Romania's 2021 edition of the OECD Country Health Profile, stroke is ranked as the second cause of mortality (with 221 deaths per 100 000 inhabitants), while the COVID-19 pandemic ranks 11^th^ (with 83.06 deaths per 100 000 inhabitants) [24, 25] (Figure 1 and Figure 2). The ranking for stroke has been maintained since 2017, when the first country report was issued, although stroke attributable deaths decreased from 18% to 16.3% [26]. As an essential tool for decision-makers and stakeholders involved in the health services provision and planning process, this publication synthesizes socio-economic, morbidity and mortality and on health system performance indicators (financing, effectiveness, accessibility, and resilience) [27].

The report highlights that stroke and COVID-19 have been the leading causes of mortality within EU-27, Norway, and Iceland [28]. In most countries, stroke was ranked as the second and third cause of death. As for deaths caused by COVID-19, it was among the top three causes of death in twenty countries.

There is considerable heterogeneity between countries regarding the percentage of total mortality. For example, COVID-19 deaths ranged from 1.1% to 15.5%, and stroke-related deaths were between 4.8% and 19.3%. Additional evidence points out that aside from COVID-19 hospital-acquired infections (3.3% in Europe and 8.4% in South America) [23], cases of stroke (as well as other neurological manifestations) [29] have been reported following laboratory-confirmed SARS-CoV-2 infection [30].

The high number of stroke cases [31] and deaths are due to modifiable (dietary, alcohol consumption, low level or lack of physical activity, and smoking) and nonmodifiable (age and sex) risk factors [32]. Behavioral risk factors are a leading cause of preventable deaths [33, 26]. Dietary risks, smoking, alcohol consumption, and low levels of physical activity are in the top five main risk factors contributing to preventable mortality and have been responsible for over 40% of the total Disability-Adjusted Life Years (DALYs) [34]. Therefore disease prevention and health promotion should be prioritized in healthcare delivery [35]. In 2014, there were 14% fewer hospital admissions referred by GPs than admissions due to a visit to the emergency department [36]. Primary [37] and secondary stroke prevention [38] reduce the burden of stroke cases and relapses as such intervention may decrease behavioral and control vascular risk factors, focusing on lifestyle changes and medication adherence [39]. The risk factor burden can be further decreased through health education and health promotion activities [40]. A quick PubMed search reveals that although the body of literature on risk factors is expanding, articles focusing on stroke prevention, health promotion, and education in countries such as Romania are scarce. Additionally, only a few studies assess the overall level of health literacy [41–43].

The COVID-19 pandemic has unquestionably played a significant role in decreasing records of stroke admissions and thus increasing stroke-related excess mortality [44] and jeopardizing access to rehabilitation for less severe cases. As both stroke and COVID-19 are public health problems with an immense burden on the healthcare system, efforts are needed on the one hand to coordinate emergency responses on all fronts to avoid endemic care disruption, and on the other hand to improve primary prevention and reverse the pyramid of service delivery, reducing overall pressure on health systems, achieving better value for money.
